# Engineering a nanoscale liposome-in-liposome for in situ biochemical synthesis and multi-stage release

**DOI:** 10.1038/s41557-024-01584-z

**Published:** 2024-07-15

**Authors:** Colin P. Pilkington, Ignacio Gispert, Suet Y. Chui, John. M. Seddon, Yuval Elani

**Affiliations:** 1 Department of Chemistry, Molecular Science Research Hub, Imperial College London, London, UK; 2 Department of Chemical Engineering, Imperial College London, London, UK

**Keywords:** Nanoscale biophysics, Self-assembly, Nanoparticles, Microfluidics

## Abstract

Soft-matter nanoscale assemblies such as liposomes and lipid nanoparticles have the potential to deliver and release multiple cargos in an externally stimulated and site-specific manner. Such assemblies are currently structurally simplistic, comprising spherical capsules or lipid clusters. Given that form and function are intertwined, this lack of architectural complexity restricts the development of more sophisticated properties. To address this, we have devised an engineering strategy combining microfluidics and conjugation chemistry to synthesize nanosized liposomes with two discrete compartments, one within another, which we term concentrisomes. We can control the composition of each bilayer and tune both particle size and the dimensions between inner and outer membranes. We can specify the identity of encapsulated cargo within each compartment, and the biophysical features of inner and outer bilayers, allowing us to imbue each bilayer with different stimuli-responsive properties. We use these particles for multi-stage release of two payloads at defined time points, and as attolitre reactors for triggered in situ biochemical synthesis.

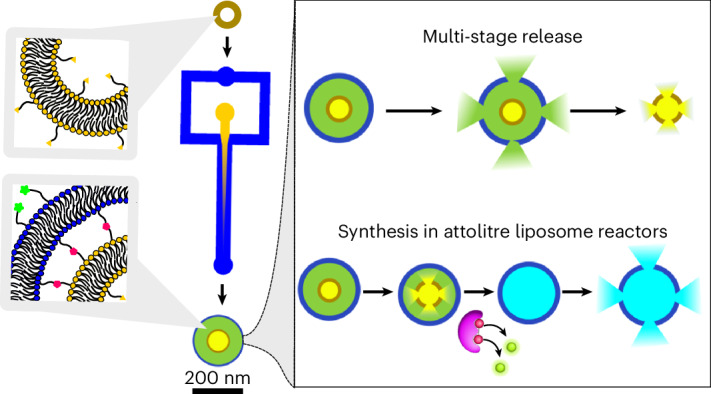

## Abstract (web summary)

Most lipid nanoparticles are structurally simplistic, existing as single-compartment assemblies. Now, a microfluidic technology to create liposomes-in-liposomes—with full control over particle features, such as the composition of each membrane, the intermembrane distance and payload of each compartment—has been developed. These particles are exploited for multi-stage release and in situ enzymatic synthesis within the particle’s attolitre volume.

## Main

Liposomes have been extensively studied in the fields of soft-matter and bottom-up synthetic biology^1–5^. As nanoparticles, they can be used as (or form part of) drug delivery vehicles^6–8^, biosensors^8,9^, chemical reactors^10,11^, synthetic organelles, and as models of biological membranes^12,13^. They are largely biocompatible and amenable to downstream functionalization^14^. However, owing to constraints imposed by existing generation methods, soft-matter nanoparticles of this type show limited morphological variation (ultimately narrowing their functional scope) and generally take the form of a single membrane-bound aqueous compartment^14^. It would be useful to simultaneously control parameters such as size, composition, and the number of lipid bilayers (lamellarity) to build lipid systems of enhanced structural complexity. This final parameter, mimicking aspects of cellular compartmentalization, is of particular interest^15–17^.

There are several examples in which multi-compartment architectures have been generated on the micrometre-scale^2,18,19^. However, applying the same approaches at the nanoscale, while maintaining a requisite level of control, is not straightforward. This is a major limitation given that particle sizes below 200 nm are required for many envisaged applications (medical or otherwise)^20^. Nanoscale nested structures (liposomes-in-liposomes) have previously been assembled using bulk hydration methods, although very little control was demonstrated over parameters needed for enhanced functionality (for example, the number of compartments and/or lamellarity, their spatial positioning, compartment dimensions, identity of encapsulated cargo, and lipid composition of each membrane)^21–27^. The production of compartmentalized nanoparticles with user-defined features would be a substantial advance. One can envisage a single particle capable of both the delivery and release of two distinct chemical species sequentially (useful for combination therapy and co-delivery of adjuvants), or indeed the synthesis of a therapeutically active compound within the particle at a defined target site, followed by release.

**Fig. 1:**
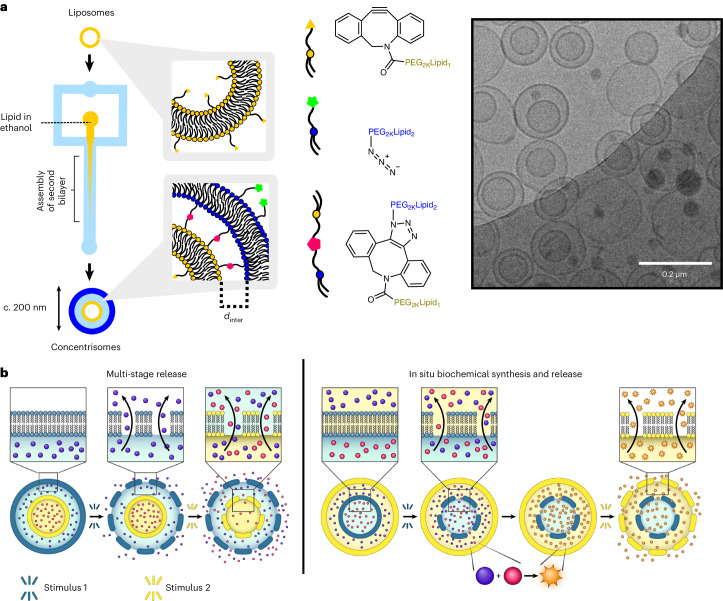
Engineering nanoscale liposomes-in-liposomes. **a**, A graphic illustrating the steps involved in the click-chemistry-assisted microfluidic synthesis of concentrisomes. A liposome suspension with alkyne moieties is fed into a MHF chip as a sheathing buffer stream, which is co-flowed alongside a secondary lipid composition that contains azide-functionalized lipids dissolved in ethanol. Micellar/bicellar aggregates tether to the external leaflet of pre-formed vesicles and eventually close to generate a second bilayer. The DBCO moiety, azide and triazole product are represented by a yellow triangle, green star and pink pentagon, respectively. The corresponding chemical structures are shown to the right of each graphic. A representative cryo-TEM micrograph of the concentrisome (double-bilayer liposome) is given on the right. Scale bar, 200 nm. **b**, A graphical illustration of possible concentrisome functionality: multi-cargo encapsulation in different compartments, triggered sequential multi-stage release of two different payloads, and in situ biochemical synthesis within the attolitre volume of the particle itself, followed by release.

We present a technology to construct compartmentalized liposomes on the nanoscale, which we term concentrisomes, using microfluidics and click chemistry (Fig. 1). The microfluidic assembly method—specifically, microfluidic hydrodynamic focusing (MHF)—proceeds via a nucleation-growth mechanism: lipids reach solubility thresholds along a well-defined diffusion gradient, nucleate and self-assemble into their most energetically favourable form. The method has been well-studied in the generation of unilamellar liposomes^14,18,28,29^. To generate concentrisomes, we introduce alternative nucleation points (pre-formed liposomes) around which new lipid bilayers assemble. We employ conjugation chemistry and PEG scaffolds to encourage preferential formation of concentrisomes and to tune the dimensions of the inter-bilayer space (*d*_inter_). We exploit our ability to control the lipid composition of each membrane to demonstrate externally triggered, multi-stage release of two small-molecule fluorescent dyes encapsulated in the inner and outer compartments of these particles. Finally, we demonstrate stimuli-responsive biochemical synthesis within the concentrisome by housing a substrate and enzyme in different compartments. Their mixing is initiated by an external trigger, leading to a reaction on the attolitre scale within the particle. Our technology has the potential to underpin new approaches for therapeutic delivery and may serve as a versatile biotechnological tool more generally.

## Results and discussion

### Concentrisome synthesis and characterization

In our initial experiments we sought to generate and characterize the base nano-architecture. To achieve this, we formed multi-compartment systems in which the outer membranes were layered upon the inner ones. Unilamellar liposomes—which would later serve as nucleation points around which additional bilayers could assemble—were first fabricated using MHF, with a flow rate ratio (FRR, defined as the aqueous flow rate divided by the ethanol flow rate) of 20 and a total flow rate (TFR) of 210 μl min^–1^. The lipid composition used was dipalmitoylphosphatidylcholine (DPPC):cholesterol:1,2-distearoyl-sn-glycero-3-phosphoethanolamine-*N*-(dibenzocyclooctyl(polyethylene glycol)-2000) (ammonium salt) (DSPE-PEG_2K_-DBCO) (53:42:5 mol%; 6.8 mM in ethanol). Dibenzocyclooctyne (DBCO), here tethered to the lipid via a PEG chain, is capable of efficient covalent bond formation with azides via a widely used 'click' reaction called strain-promoted azide/alkyne cycloaddition (SPAAC). A PEG_2K_ spacer in a brush conformation (5 mol%) was included for two reasons: (1) to distance the site of conjugation away from the lipid bilayer, and (2) to define the dimensions of *d*_inter_, noting the precedent for PEG-mediated bilayer spacing^30,31^. This nanoparticle suspension was then introduced into a second MHF chip such that it sheathed a central ethanolic stream, containing lipids of composition DPPC:cholesterol:1,2-distearoyl-sn-glycero-3-phosphoethanolamine-*N*-(azido(polyethylene glycol)-2000) (ammonium salt)(DSPE-PEG_2K_-N_3_) (53:42:5 mol%; 6.8 mM in ethanol). An FRR of 20 was chosen again with a TFR of 210 μl min^–1^.

**Fig. 2:**
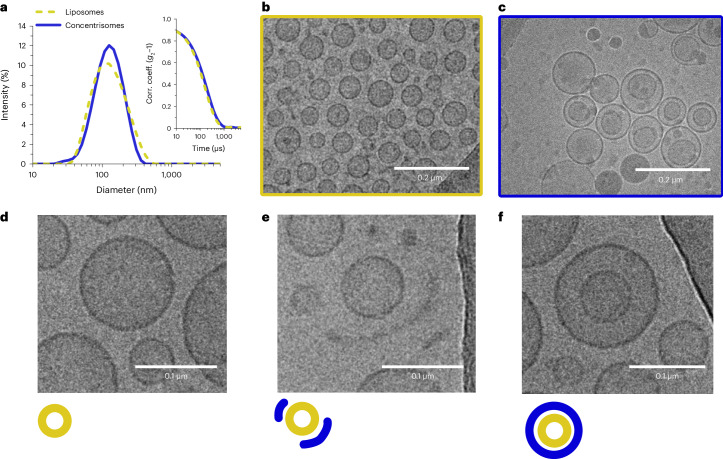
Characterization of concentrisomes via DLS and cryo-TEM. **a**, Dynamic light scattering data shows a slight shift of the hydrodynamic diameter of input liposomes, functionalized with DBCO, from approximately 100 nm (PDI = 0.16) to 120 nm (PDI = 0.26), after exposure to a new lipid composition via an MHF chip containing azide-functionalized lipids. Correlograms (correlation coefficient (*g*_2_−1) versus time) for both Gaussians are shown in the inset. **b**, Representative cryo-TEM micrograph for input liposomes (yellow), which are unilamellar. **c**, Representative cryo-TEM micrograph for concentrisomes (blue), showing inner and outer compartments. **d**–**f**, A graphic illustrating the potential stages of concentrisome growth with corresponding cryo-TEM examples. Left to right: pre-formed liposomes (**d**) act as nucleation points in the MHF chip, around which bicelle/micelles with azide functionality begin to assemble (see central micrograph in **e**). We propose that these covalently tethered assemblies eventually close over to generate an external spherical bilayer, generating the concentrisome morphology (**f**). Source data

Dynamic light scattering (DLS) was used to assess the size of each population and track the formation of higher-order structures. A slight increase in the mean diameter was observed from approximately 100 nm to 120 nm (PDI = 0.16 and 0.26, respectively)—taken to indicate possible growth of a second bilayer (Fig. 2a). Each particle population was imaged using cryo-TEM where a clear difference in particle lamellarity could be observed (Fig. 2b,c). Estimates from particle counting over 50 micrographs indicated the presence of double-bilayer vesicles at 60% of the total particle population (see Supplementary Fig. 1 for additional micrographs). Importantly, no vesicles with more than two bilayers were observed. The co-existence of smaller structures (thought to indicate the presence of either micelles or PEGylated bicelles) could occasionally be seen by DLS (~1–30 nm). These structures were directly imaged using cryo-TEM (Supplementary Fig. 2) and are understood to be a common by-product of PEGylated-lipid/DPPC binary mixtures, taking the form of flat disks composed of a single lipid bilayer^32,33^. A tentative mechanism for concentrisome formation was proposed: that micellar and/or bicellar structures conjugate via click chemistry to pre-formed vesicles, eventually closing to form an additional bilayer. This was supported via cryo-TEM: some vesicles were observed to be surrounded by curved bicelle portions, thought to represent an intermediate state between unilamellar and concentrisome morphologies (Fig. 2d–f).

### Mechanistic insights and process optimization

We investigated the role of click chemistry in concentrisome formation. First, we ran the concentrisome synthesis procedure as before, but replaced terminally functionalized alkyne lipids with DSPE-PEG_2K_, such that the lipid composition used in both stages was DPPC:cholesterol:DSPE-PEG_2K_ (53:42:5 mol%; 6.8 mM). Particle suspensions were analysed using DLS and cryo-TEM. Although some double-bilayer vesicles were observed, the majority of particles remained unilamellar, with under 10% of the total population exhibiting an additional bilayer (Supplementary Fig. 3). This was taken to infer a positive covalent click interaction between terminally functionalized PEG spacers promoted the formation of concentrisomes.

We then used the fluorogenic and membrane-permeable dye 3-azido-7-hydroxycoumarin to probe the reactivity of DBCO (ring-strained alkyne) functional groups before and after concentrisome formation. The dye is capable of SPAAC chemistry and, after generating a triazole with ring-strained alkynes, produces a fluorescent signal^34^. It was proposed that the number of available alkynes would reduce following the assembly of an additional bilayer via SPAAC around the pre-formed liposomes, leading to a lower intensity fluorescent signal. The concentration of DSPE-PEG_2K_-N_3_ in the outer bilayer was varied from 0 to 5 mol%, off-set by DSPE-PEG_2K_, such that the total PEGylated-lipid concentration was equal throughout. The particle concentration of the inner-alkyne-functionalized liposomes, and the flow rates used, were kept constant (FRR = 20; TFR = 210 µl min^–1^). We added 3-azido-7-hydroxycoumarin (with a final concentration of 6.15 μM) to each sample and analysed the emergence of a fluorescent signal over time. Results are shown in Supplementary Fig. 4b where an exponential decay in the fluorescence intensity for the triazole was observed as a function of azide-lipid present in the second bilayer. From this it was concluded that terminally functionalized DSPE-PEG_2K_ alkyne lipids were interacting via SPAAC chemistry.

Our assay was extended to explore the role of the (1) FRR and (2) TFR on the formation of concentrisomes via SPAAC. A lower fluorescence intensity for the triazole–coumarin adduct was taken as a proxy for the efficiency of concentrisome formation. The FRR values were varied from 10 to 60 and azidocoumarin was added to each sample as before. A second population at each FRR value was generated—this time using DSPE-PEG_2K_ instead of DSPE-PEG_2K_N_3_—to serve as an internal reference from which the fluorescence intensity of the 'click' population could be compared (producing a change in fluorescence intensity Δ*I*). Under these assumptions, a larger Δ*I* corresponded to a more efficient SPAAC reaction, and, by extension, concentrisome formation. The TFR was varied from 200 to 450 µl min^–1^, and the assay (with internal references) was conducted as before (Supplementary Fig. 4c). We found that an FRR value of 20, as previously used, yielded the largest *ΔI* value. There was no clear dependence between concentrisome formation efficiency and TFR, and 210 µl min^–1^ remained the TFR of choice for future experimentation.

### Using PEGylated lipid to modulate *d*_inter_

**Fig. 3:**
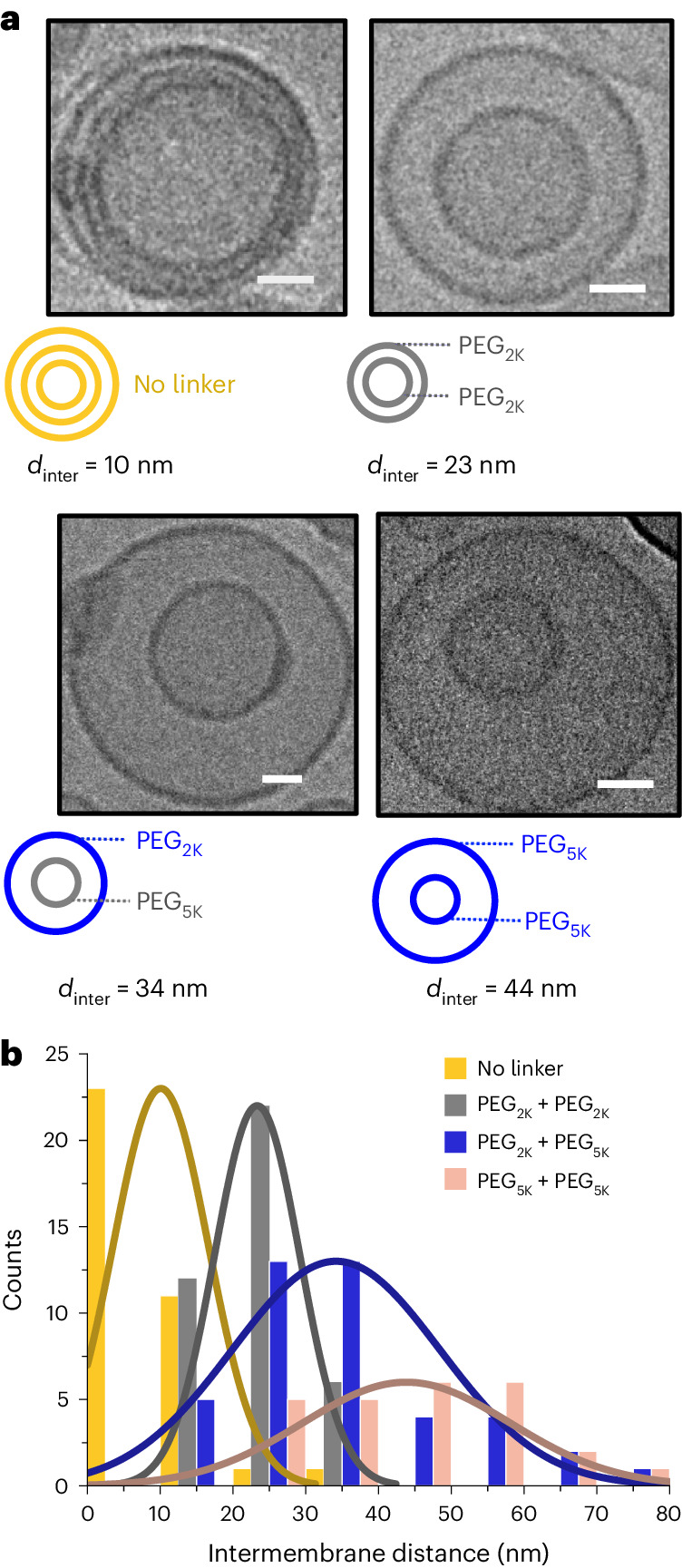
Relationship between PEGylated-lipid length and intermembrane space. **a**, The variation of *d*_inter_ between successive bilayers, using PEG chains of different molecular weights, and in various combinations, is shown. Micrographs correspond to combinations, with average *d*_inter_ values of 10, 23, 34 and 44 nm, for no-linker, PEG_2K_DBCO:PEG_2K_N_3_, PEG_2K_DBCO:PEG_5K_N_3_ and PEG_5K_DBCO:PEG_5K_N_3_, respectively. Scale bars, 25 nm. **b**, A histogram with measured *d*_inter_ values for each linker combination. Gaussian plots are added for clarity. A gradual increase in *d*_inter_ is seen with increasing combined PEG length; >25 concentrisomes were analysed to generate each distribution. Source data

Cryo-TEM was used to provide a more detailed structural analysis of the particles we generated, in particular, the dimensions of *d*_inter_. It was proposed that this dimension could be modulated using PEG spacers with varying persistence lengths. To explore this, concentrisomes containing PEGylated lipids of different PEG lengths were generated under the same conditions as before; *d*_inter_ was measured for each concentrisome. A point at the approximate centre of each concentrisome was chosen and the annular space calculated in four directions (Supplementary Fig. 5). When non-PEGylated liposomes without azide/alkyne functionality were generated, evidence of a higher percentage of multi-lamellar structures was observed, although the number of bilayers per particle was varied (average *d*_inter_ ≈ 10 nm). On the other hand, clear two-compartment liposomes were observed when using PEG-tethered 'clickable' groups, with *d*_inter_ increasing with the length of the PEGylated lipid of the inner and outer bilayers (histogram and cryo-TEM snapshots are shown in Fig. 3a,b). For a PEG_2K_ DBCO/PEG_2K_ azide combination, *d*_inter_ averaged at 23 nm. For a PEG_2K_ DBCO/PEG_5K_ azide combination, the average was 34 nm, and a PEG_5K_ DBCO/PEG_5K_ azide combination averaged at 44 nm. This notable increase was taken to infer that PEG tethers were present in a largely uncoiled brush conformation perpendicular to each lipid bilayer, acting as steric scaffolds at 5 mol% (ref. ^35^).

### Controlling bilayer composition

**Fig. 4:**
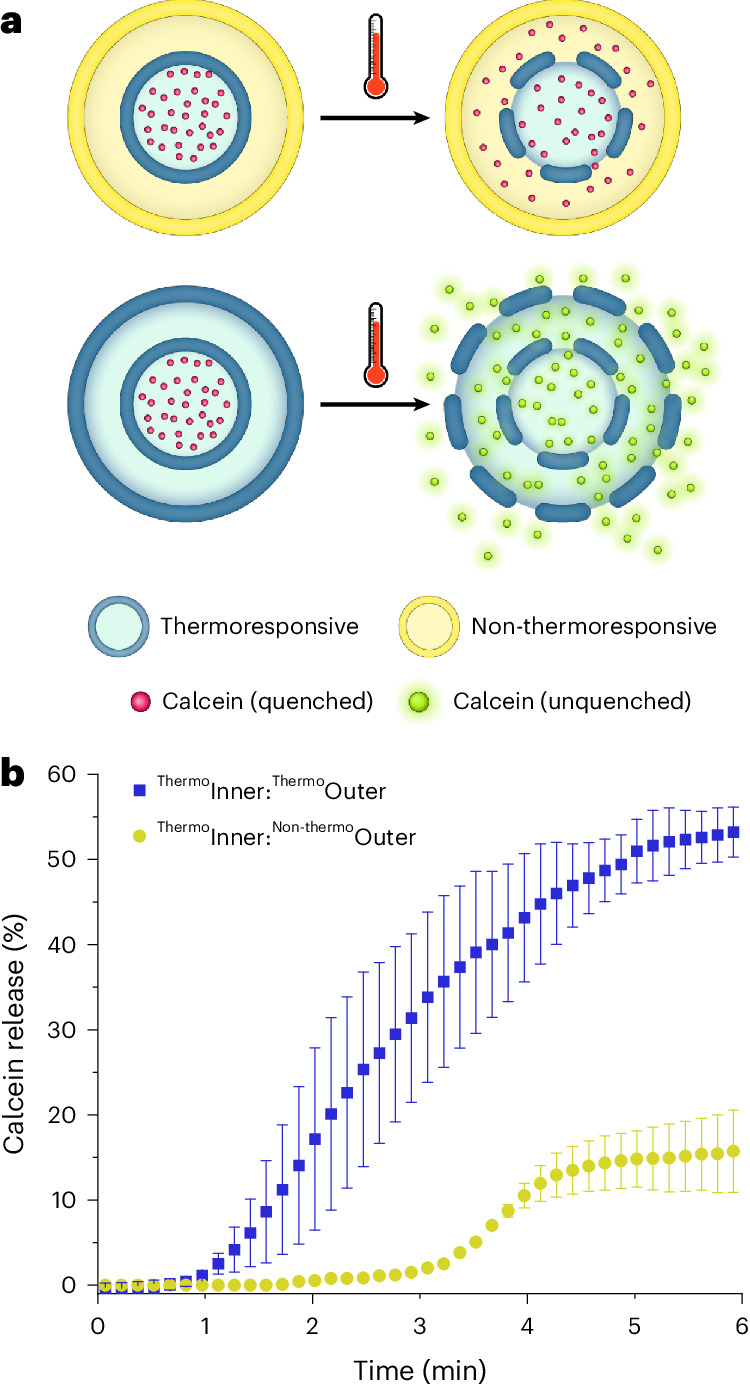
Validation of layering and compositional control. **a**, A graphic illustrating the main events of the assay, showing the ability to control the composition of each bilayer (in this case making them thermoresponsive and non-thermoresponsive). Initially the dye is quenched (non-fluorescent). For concentrisomes with a non-thermoresponsive outer bilayer, calcein was not expected to undergo sufficient dilution via efflux to unquench and produce a fluorescent signal. **b**, Percent calcein release profiles at 42 °C for each population. When both membranes were thermoresponsive, the fluorescence increase was much higher than when only the inner one was, because, in the latter, calcein remained encapsulated in a small enough volume to prevent unquenching. The release profiles for the additional controls mentioned in the main text can be found in Supplementary Fig. 6, whereas compositional details can be found in Supplementary Fig. 7. Values were calculated from fluorescence intensities after addition of surfactant (Triton X-100, 5 wt%, 2.5 µl min^–1^). Error bars indicate the s.d. of the average intensities for *n* = 3. The <20% calcein release for ^**Thermo**^**Inner:**^**Non-thermo**^**Outer** concentrisomes was thought to originate from unilamellar vesicles that had not undergone click chemistry. Source data

A fluorescence assay was developed to demonstrate the potential for concentrisomes to act as compartmentalized, multifunctional nano-capsules, and that the composition of each bilayer can be user-defined. The assay used the self-quenching dye calcein and a thermoresponsive lipid composition capable of cargo release following heating to a gel/fluid phase transition temperature (*T*_m_). We were able to develop a system where one bilayer was thermoresponsive and the other was non-thermoresponsive (graphically illustrated in Fig. 4a; a full list of each composition used is given in a table as part of Supplementary Fig. 7).

The dye was loaded at self-quenching concentration (40 mM) into thermoresponsive liposomes (DPPC:cholesterol:DSPE-PEG_2K_-DBCO; 6.8 mM; 90:5:5 mol%), represented in Fig. 4a as a blue circle. For clarity, this composition was termed ^**Thermo**^**Inner**. A suspension of these liposomes was exposed to a second lipid composition via MHF, a non-thermoresponsive composition denoted ^**Non-thermo**^**Outer** (DPPC:cholesterol:DSPE-PEG_2K_-N_3_; 6.8 mM; 53:42:5 mol%), represented as a yellow bilayer in Fig. 4a, with no dye present in the intermembrane space. The resulting concentrisome composition was termed ^**Thermo**^**Inner:**^**Non-thermo**^**Outer**. Upon heating to 42 °C a gel-to-fluid phase transition was initiated for ^**Thermo**^**Inner**, coupled with the release of calcein from the inner compartment to the lumen of the surrounding non-thermoresponsive liposome. The outer bilayer was expected to retain any released calcein in a volume such that the dye remained at a self-quenched concentration.

A number of control populations were prepared. The first was a scenario in which both inner and outer bilayers were thermoresponsive, leading to full cargo release at elevated temperatures. These concentrisomes were formed using the same flow conditions and sheathing stream liposomes (^**Thermo**^**Inner**) as before. The first had an outer bilayer consisting of ^**Thermo**^**Outer** (DPPC:cholesterol:DSPE-PEG-N_3_; 6.8 mM; 90:5:5 mol%) where both bilayers of the concentrisome were rendered thermoresponsive (denoted ^**Thermo**^**Inner**^**Thermo**^**Outer**). The release profiles, shown in Fig. 4b, revealed a considerable decrease in percent calcein release for concentrisomes with a non-thermoresponsive outer bilayer, indicating that a new bilayer of a different composition had successfully formed around pre-existing liposomes.

Incorporating cholesterol into membranes modulates their thermoresponsive properties. To address the possibility that enough cholesterol was unintentionally inserted into the inner bilayer during recirculation, enough to alter the *T*_m_ of ^**Thermo**^**Inner** to render it non-thermoresponsive and producing a false positive, we ran an experiment in which only cholesterol was present in the recirculating ethanol stream, at a concentration equivalent to that used in our previous experiment (2.86 mM). No change in the calcein release profile was noted for these liposomes (see Supplementary Fig. 6b). We also generated a concentrisome population with a fluid phase outer bilayer (that is, non-thermoresponsive) without cholesterol, consisting of DOPC:DSPE-PEG_2K_-N_3_ (6.8 mM; 95:5 mol%, ^**DOPC**^**Outer**). The calcein release profile of this final control (termed ^**Thermo**^**Inner**:^**DOPC**^**Outer**) resembled that of ^**Thermo**^**Inner:**^**Non-thermo**^**Outer** (Supplementary Fig. 6d), once again suggesting that a change in calcein release could not be attributed to cholesterol migration between bilayers.

Finally, to further examine whether formation of concentrisomes was aided by covalent linkage between bilayers (building on previous experiments) we ran another control using our ^**Non-thermo**^**Outer** composition without azide-functionalized lipids (DPSE-PEG_2K_). Calcein release for this concentrisome system (termed ^**Thermo**^**Inner**:^**PEG**^**Outer**) resembled that of ^**Thermo**^**Inner**:^**Thermo**^**Outer**, further supporting our theory that concentrisome formation was promoted by covalent linkages between successive bilayers (see Supplementary Fig. 6c).

These results allowed us to reach a number of key conclusions about the concentrisome system: (1) the outer bilayer composition could be tuned to have different physical and stimuli-responsive properties to the inner bilayer; (2) click chemistry and a covalent linkage between opposing bilayers is necessary for concentrisome synthesis; and (3) cholesterol, under these conditions, does not passively insert into pre-formed vesicles (at least enough to alter the *T*_m_ of the inner bilayer).

### Multi-stage release

**Fig. 5:**
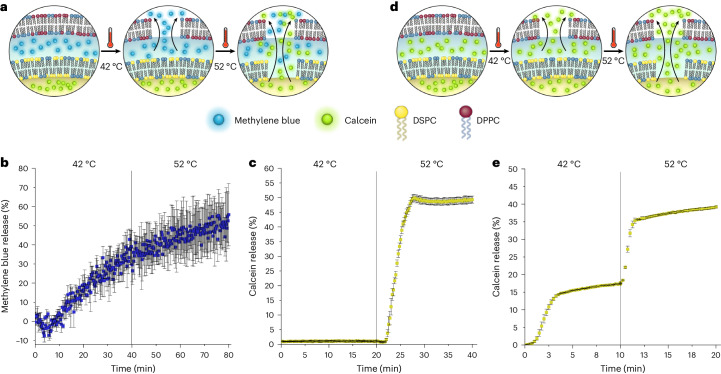
Multi-stage payload release. **a**, A graphic illustrating multi-stage sequential release of two different cargos from two concentrisomes compartments. The two cargos are self-quenching dyes and thus release leads to an increase in fluorescence. **b**, Percent release of methylene blue over time from the concentrisome system with outer and inner bilayers composed of DPPC and DSPC, respectively. As methylene blue was isolated to the intermembrane space, the dye was released at 42 °C (above the transition temperature of the outer membrane DPPC composition). Data are represented as mean values ± 1 s.d. for *n* = 3. **c**, Percent calcein release over time for the same concentrisome system. In this case calcein was isolated to the inner liposome, thus release was observed only at 52 °C (which is above the transition temperature of this DPSC composition, the main component of the inner bilayer). Data are represented as mean values ± 1 s.d. for *n* = 3. **d**, A graphic illustrating the events of a multi-stage sequential release of the same cargo (calcein dye) from the two compartments of a concentrisome. Calcein was encapsulated in both the inner liposome and intermembrane space of a concentrisome system, with the inner bilayer composed of our DSPC composition (*T*_m_ ≈ 52 °C), and the outer bilayer of our DPPC composition (*T*_m_ ≈ 42 °C). **e**, Percent release of calcein dye over time, showing two discrete bursts when the samples are heated to the phase transition temperatures of the inner and outer membranes. Data are represented as mean values ± 1 s.d. for *n* = 3. Further controls can be found in Supplementary Fig. 8. Source data

An additional fluorescent assay was developed to show that concentrisomes were capable of encapsulating multiple different payloads in different compartments, each released sequentially via a defined external stimulus (see Fig. 5a for a graphical illustration). In these experiments, two different dyes were encapsulated in the outer (methylene blue) and inner (calcein) compartments. Using a combination of multi-angle DLS measurements and fluorescence calibration, approximate encapsulation efficiencies were determined for (1) calcein in the inner core of the concentrisomes, and (2) methylene blue present in the intermembrane space (see Supplementary Fig. 9). By employing different lipids, the outer and inner membranes were designed to release at lower and higher temperatures, respectively. Sequentially exposing our sample to a low temperature followed by a high temperature would lead to the release of methylene blue first, then calcein.

To test this, thermoresponsive liposomes with composition DSPC:cholesterol:DSPE-PEG_2K_-DBCO (1 mM; 80:15:5 mol%), termed ^**Thermo**^**Inner 2** (*T*_m_ ≈ 50 °C), were generated using MHF, with encapsulated calcein (40 mM). Once purified, these liposomes were subjected to the MHF layering method and introduced to their azide counterpart in the form of ^**Thermo**^**Outer** (*T*_m_ ≈ 42 °C) with methylene blue dissolved at 40 mM in the sheathing stream. This was done to encapsulate the dye within the intermembrane volume. A second purification step was required to remove excess methylene blue. The concentrisome population was denoted ^**Thermo**^**Inner 2:**^**Thermo**^**Outer**. A number of control populations were generated. Control 1 was produced in the same way (with both calcein and methylene blue), this time using DPPC-based thermosresponsive compositions for both the inner and outer bilayers, that is, ^**Thermo**^**Inner:**^**Thermo**^**Outer**. Control 2 reversed the order of the DSPC and DPPC compositions giving ^**Thermo**^**Inner:**^**Thermo**^**Outer 2**. Finally, control 3 had the composition ^**Thermo**^**Inner 2:**^**Thermo**^**Outer**, this time replacing methylene blue with additional calcein (40 mM).

The release profile for methylene blue for the concentrisomes in our test system, ^**Thermo**^**Inner 2:**^**Thermo**^**Outer**, was analysed at 42 °C and at 52 °C. As expected, a ~40% release of methylene blue was observed at the lower temperature, which corresponded to release of the dye from the outer compartment through the outer DPPC-based membrane (Fig. 5b). The release profile for encapsulated calcein was then analysed (using a fresh concentrisome sample) at 42 °C and 52 °C; ~50% calcein release was observed only at the higher temperature (see Fig. 5c), in accordance with the *T*_m_ value of our DSPC composition.

Using the same concentrisome composition, we also demonstrated that it is possible to achieve multi-stage, sequential release of the same payloads in two discrete bursts by loading calcein in both the inner and outer compartments (see Fig. 5d,e). Here we observed two sharp increases in fluorescent signals, corresponding to release events: the first upon reaching the phase transition temperature of the outer membrane (42 °C) and the second upon reaching that of the inner membrane (52 °C).

All control populations followed the expected order of release (see Supplementary Fig. 8), demonstrating it was possible to simultaneously control both successive bilayer composition and location of multiple encapsulants within a concentrisome. From these results we concluded that discrete cargos can be encapsulated in distinct compartments within the same concentrisome, and that two defined stimuli can be used to release those cargos.

### Compartmentalized biochemical synthesis

Having demonstrated the ability to sequentially release multiple encapsulants, we went on to develop a compartmentalized system capable of the controlled mixing of an enzyme and substrate within a single particle, by defining the physical properties of each successive bilayer as before. The system chosen was that of the β-galactosidase (β-Gal)-mediated hydrolysis of non-fluorescent fluorescein di-β-d-galactopyranoside (FDG) to produce fluorescein (fluorescent)^2^. In these experiments, the enzyme and substrate would initially be housed in different compartments, with the particle being dormant. Only upon permeabilization of the inner compartment would the substrate meet the enzyme, leading to a chemical reaction confined in the lumen of the outer compartment.

**Fig. 6:**
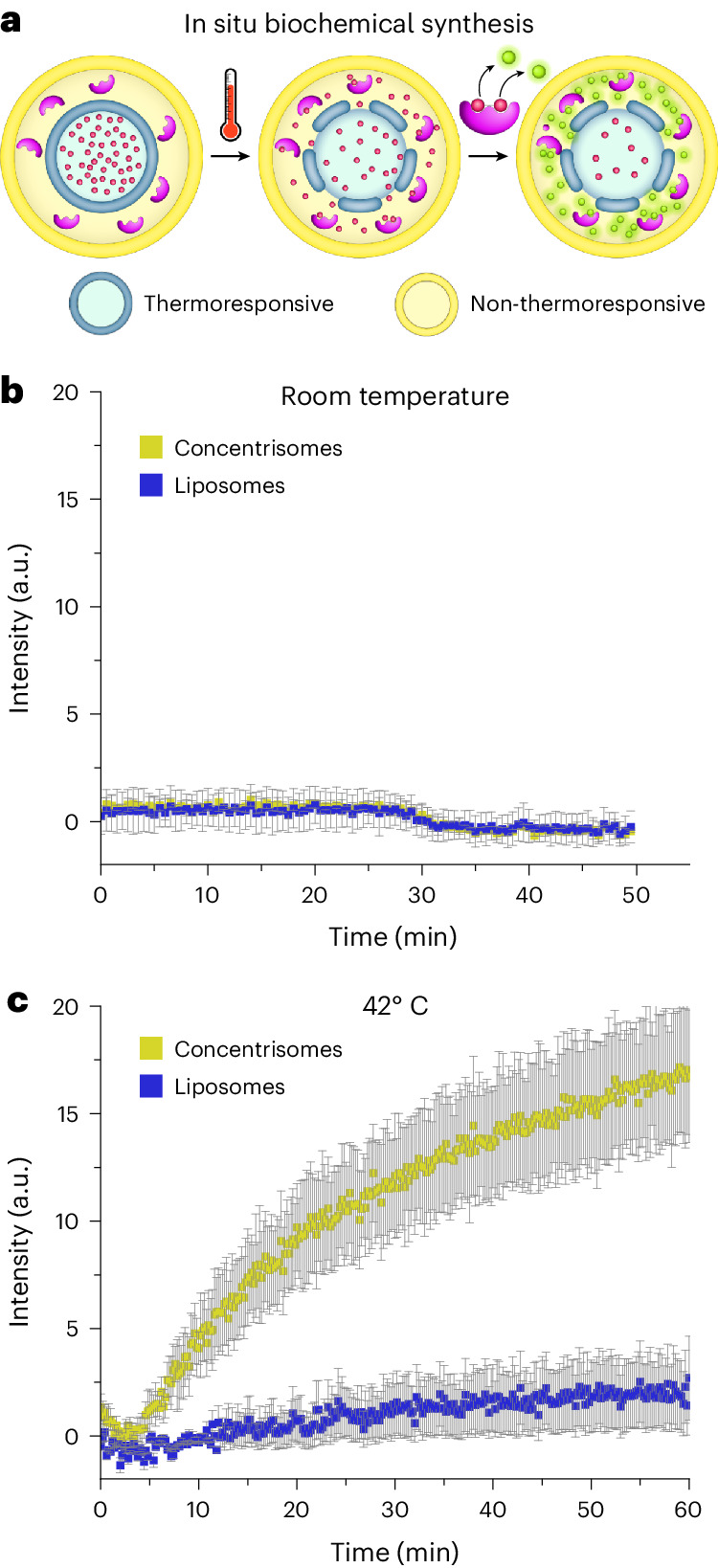
Triggered biochemical synthesis within the nanoparticle. **a**, Graphic illustrating in situ enzymatic synthesis in attolitre concentrisome reactors. Thermoresponsive liposomes containing the FDG substrate were recirculated with non-thermoresponsive lipids and β-Gal using an MHF flow regime identical to the flow conditions as before. In the control, liposomes were recirculated with ethanol and β-Gal only. **b**, Fluorometric data tracking the hydrolysis of FDG to fluorescein (*λ*_ex_ = 498 nm; *λ*_em_ = 517 nm) at room temperature. No fluorescence increase was observed indicating enzyme and substrate remained compartmentalized. **c**, The same populations at 42 °C, showing an increase in fluorescence in the concentrisome sample. This indicates the thermoresponsive inner compartment permeabilizes, leading to the content mixing and subsequent enzymatic reaction occurring within the concentrisome. Error bars indicate the s.d. of the mean between three separate experiments, for both control (blue) and test (yellow) results. Further control experiments can be found in Supplementary Figs. 10–12. Source data

The substrate FDG (0.15 mM) was first encapsulated in thermoresponsive liposomes (^**Thermo**^**Inner**) formed using extrusion, and then purified by size exclusion chromatography. As before, these liposomes where then fed through the MHF chip via the aqueous sheathing stream. This was performed in the presence of β-Gal (0.5 U ml^−1^) in the aqueous stream and ^**Thermo**^**Outer** lipids dissolved in the ethanol stream, trapping the enzyme between the two bilayers (Fig. 6a). Trypsin (0.25 wt%) was added to collected samples to catalyse the proteolysis of unencapsulated β-Gal and prevent a false positive (that is, bulk hydrolase activity). The effect of trypsin on the bulk enzymatic reaction is shown in Supplementary Fig. 10. A sample of single bilayer liposomes of ^**Thermo**^**Inner** composition containing FDG was used as a control (trypsin was added to the bulk medium).

We monitored the production of fluorescein first at room temperature, and then at 42 °C. We observed negligible enzyme activity at room temperature for both the concentrisome system and single bilayer liposome control (Fig. 6b). However, raising the temperature and crossing the gel/fluid transition of the inner bilayer (containing the FDG substrate) led to an increase in fluorescence intensity for the concentrisome population (Fig. 6c). The fluorescent product was largely isolated to the lumen of the concentrisome system and could be collected using centrifugal filtration (see Supplementary Fig. 11). The single bilayer liposome control showed a negligible increase with all unencapsulated β-Gal degraded by trypsin. From this we inferred that enzyme activity was isolated solely to the double-bilayer system. This was supported by an additional control, detailed in Supplementary Fig. 12, where the enzyme and substrate were encapsulated in two separate unilamellar vesicle populations and then mixed. Even at an elevated temperature, no fluorescein product was detected.

## Conclusion

We have developed a microfluidics-based technology that allows the assembly of designer, nanoscale, multi-compartment liposomes by controlling key architectural and compositional parameters. This in turn has allowed us to engineer particles capable of multi-stage release and stimuli-responsive enzymatic synthesis within the particle. Existing methodologies do not grant this level of control over: (1) bilayer composition, (2) intermembrane distance, (3) the positioning and identity of the encapsulants, and (4) their overall size. We expect this technology to have applications in biotechnology and beyond, including in drug and vaccine delivery.

Multi-stage release of different cargoes can be beneficial in combination therapies, enabling the sequential release of varied payloads with complementary effects in the same location enabling coordinated responses. Furthermore, releasing the same cargo at different time points can be advantageous for accurate dosing and controlled pharmacokinetics, ultimately leading to improved therapeutic windows. Moreover, on-site synthesis of a therapeutically active compound inside a delivery vehicle in close proximity to a target could increase site selectivity, conferring all of its associated benefits.

Performing chemistry inside attolitre reaction vessels is generally attractive, especially when dealing with scarce or expensive reactants, as well as enabling mass parallelization of reactions and high-throughput screening. A considerable reduction in size can bring about additional challenges (mainly related to characterization) but ultimately renders these particles more appropriate for use in biological systems, where access through sub-micrometre constrictions (for example, in capillaries and tumours) is required^21^.

We note that the microfluidic generation methods and broad compositions used in this work are similar to those commonly employed for liposome and lipid nanoparticle synthesis in clinical settings. Furthermore, we do not anticipate limitations regarding the incorporation of different lipid types. Lipids used in standard, clinically approved liposomes are expected to be compatible with our systems as well. We therefore expect our system to be amenable to compositional adjustments, allowing optimization of our particles for biomedical applications, particularly in terms of uptake, endocytosis, endosome escape, clearance and immune evasion properties^36^.

This work also has implications for bottom-up synthetic biology, where vesicle-based artificial cells are used as bioinspired microdevices, compartmentalized reactors and cellular models^37,38^. Our double-membrane systems are biomimetic in that they approximate many membranous architectures in cells composed of several layered bilayers with similar spatial dimensions (for example, bacterial double membranes^39^, organelle membranes^40^, gap junctions^41^). Our particles could be used in this context, acting as reconstitution scaffolds for these cellular motifs.

Importantly, the on-chip nature of our technology allows size control and scalable continuous manufacture of particles in a continuous manner, in high-throughput, with reduced sample and reagent requirements^14,28^. It will also allow integration with other lab-on-chip modules for downstream processes (for example, purification^42^ and sterilization^43^) in follow-up studies.

In summary, enhanced control offered by microfluidic methods and careful composition choice has allowed us to finely tune intermembrane volume, and, crucially, spatially segregate encapsulants. We offer a potential mechanism to explain the formation of particles that is two-fold: nucleation-growth of a new bilayer is instigated around pre-formed single bilayer liposomes, aided by a covalent interaction, in the form of a 1,3 dipolar cycloaddition, between the terminal groups of PEG functionalized lipids doped within each bilayer. We demonstrate that each bilayer can be imbued with different physical properties, here exemplified in a thermo/non-thermoresponsive system. This functionality alone has profound implications for future drug delivery technologies, particularly where multiple therapeutics are required. The system is highly adaptable and could be extended to include other amphiphiles (lipid and/or polymer) with unique stimuli-responsive properties (ultraviolet, pH, magnetism for example)^44,45^.

Future work will focus on enhancing the architectural complexity of these constructs and the development of assays relevant to current pharmaceutical goals (the encapsulation of genetic material and accompanying adjuvants, and so on). Terminal azide groups could in theory be used to form additional bilayers, allowing the synthesis of structures with >2 bilayers. The versatility of our system will also facilitate purification strategies: one could exploit the functional aspect these particles to perform column chromatography^46^, or size-based separation techniques. Finally, we hope that this work will inspire others to continue to widen the functional landscape of drug delivery vehicles by focusing on alternative lipid architectures.

## Methods

### Microfluidic chip fabrication

Polydimethylsiloxane (PDMS) chips used to perform MHF experiments were fabricated using established photolithography techniques^21^. SU-8 photoactive polymer (3050; Micro Resist Technology) was spin coated onto a silicon wafer (1,500 r.p.m.; 30 s; WS-650-8 model, Laurell Technologies Corporation), creating a relatively even film. This was heated to 95 °C for 45 min on a hotplate. A laser cut photomask with desired features was carefully placed on the photoactive polymer film and exposed to ultraviolet light (9.7 mV; 300 mJ cm^–^^2^ dose), before being heated to 65 °C (5 min) and 95 °C (12 min). Excess photoresist was washed away using propylene glycol methyl ether acetate (Sigma-Aldrich), and finally with isopropyl alcohol. The wafer was heated to 120 °C for 1 h before being coated with a thin layer of perfluorooctyl triethoxysilane in a desiccator. A vigorously mixed 10:1 mixture of PDMS:curing agent was then added to this mould. The polymer was degassed in a desiccator and then cured at 65 °C for 8 h. Chips were then cut from the cured PDMS and bonded to a flat slab of PDMS using air plasma, closing the microfluidic channels (around 150 μm × 150 μm in width and depth). The .svg file is available on request from the corresponding author.

### Formation of concentrisomes via MHF

All lipids were purchased from Sigma-Aldrich (Avanti Polar Lipids). Unilamellar vesicles were first generated via MHF. A lipid film containing the lipid mixture (DPPC:cholesterol:DSPE-PEG(XK)-DBCO at 53:42:5 mol%) was dissolved in ethanol to generate a 6.8 mM solution. Bath sonication was necessary to break down lipid aggregates. This solution was injected into the central channel of an MHF chip, and sheathed by buffer streams composed of HEPES/KCl (150 mM; 20 mM at pH 7.4). The FRR was 20 (unless otherwise specified), with a TFR of 210 µl min^–1^. The resulting particle suspension was then used as the sheathing buffer for a separate MHF chip, where DPPC:cholesterol:DSPE-PEG(XK)-N_3_ (6.8 mM; 53:42:5 mol%) was used as the central stream. The same flow conditions were used. Samples were collected and analysed without further purification. For fluorescent dye encapsulation (either calcein or methylene blue) the required dye concentration was dissolved in the buffer streams (calcein = 40 mM; methylene blue = 40 mM). To separate concentrisomes and/or liposomes from unencapsulated dye, particle suspensions were then purified using a Sephadex G-50 size exclusion chromatograph (5 ml; HEPES/KCl/glucose at 150 mM, 20 mM and 0.5 M at pH 7.4, respectively) and appropriate fractions were stored at 4 °C to await further use. Compositional details (as specified in the main text) can be found in Supplementary Fig. 7.

### DLS

Hydrodynamic diameters and polydispersity indices of vesicular structures formed were approximated using DLS (Malvern Zetasizer Ultra). Samples were diluted to 10:1 in deionized water before analysis. Malvern software-generated correlograms and laser auto-attenuation values were used to judge the reliability of the Gaussian plots obtained. Samples were run in triplicate to account for any measurement variations. Multi-angle dynamic light scattering (Malvern Zetasizer Ultra) was occasionally used to estimate the particle concentration (in particles per millilitre) of the vesicle suspensions. A background scattering count of 80 kcaps was used for the experiment detailed in Supplementary Fig. 9.

### 3-Azido-7-hydroxycoumarin assay

A membrane-permeable fluorogenic dye 3-azido-7-hydroxycoumarin (Broadpharm) was used to qualitatively assess the concentration of free DBCO groups before and after microfluidic layering of a second bilayer, and to optimize microfluidic flow conditions. Following the formation of a triazole ring via SPAAC chemistry with strained alkynes, a fluorescent signal is produced^36^. A solution of the dye in 1 × PBS at pH 7.4 was added to particle suspensions at 6.15 μM. Samples were agitated before immediately tracking the emergence of a fluorescent signal with *λ*_abs_ = 404 nm; *λ*_em_ = 477 nm. Analysis was performed in triplicate (three separate experiments).

### Cryo-TEM

Cryo-TEM was used to assess the size and morphology of vesicular structures, in particular, the lamellarity of each structure. Collected samples were analysed without further treatment; 3.5 μl of the sample was transferred to a plasma-treated carbon grid (Quantifoi R 2/2 on 300 copper mesh; Jena Bioscience) in a Vitrobot (Thermo Fisher Scientific) at 95% humidity. The vesicles were allowed to deposit for 35 s. The grid was then blotted (blot force = 0; blot time = 7 s) and quickly plunged into a liquid ethane bath that was kept at approximately –180 °C. Grids were transferred under liquid nitrogen to an electron microscope sample holder (Cryo Transfer Tomography holder, Eden Instruments; Model 2550). Defocus values (generally between –0.5 and –5 µM) were determined elsewhere on the grid to avoid sample damage from the electron beam. We used a FEI Tecnai12 bio twin 120 kV with a TVIPS XF416 4 K CMOS detector. Image handling and analysis was performed using ImageJ software^47^. Particle counting was performed manually.

### Calcein release assay

Thermoresponsive vesicles containing calcein (40 mM) and decorated with DBCO groups were transformed into concentrisomes as described above. Samples were analysed in 4.5 ml disposable cuvettes using a CARY ultraviolet–visible/fluorescence spectrometer. To initiate the phase transition of the inner compartments from L_β_ to L_α_, cuvettes containing HEPES/KCl/glucose (1,300 μl; 150 mM, 20 mM and 0.5 M at pH 7.4, respectively) were heated externally via a Peltier block. A digital thermometer was used to confirm the buffer had reached the desired temperature, at which point vesicle samples (200 μl) were added, and fluorescence intensity at *λ*_ex_ = 495 nm; *λ*_em_ = 517 nm measured over time. Percent calcein release values were then calculated from intensities read after vesicle micellization with Triton X-100 (2.5 μl; 5% w/v) (Sigma-Aldrich).

### Multi-stage release assay

Unilamellar vesicles with thermoresponsive bilayers (composition B_DBCO_) containing calcein (40 mM) were generated and purified as previously described. Methylene blue (40 mM) was then dissolved in this vesicle suspension and transformed into concentrisomes with varying outer bilayer compositions (see main text for details). The resulting concentrisomes were purified by size exclusion chromatography a second time to remove unencapsulated methylene blue and analysed via fluorescence spectroscopy.

### β-Gal/FDG assay

Fluorescein di-β-d-galactopyranoside (0.15 mM) was encapsulated in thermoresponsive vesicles with DBCO groups via extrusion, as described above, and purified by size exclusion chromatography. Samples were diluted 10:1 in a HEPES/KCl buffer containing β-Gal (0.5 U ml^–^^1^) and then used as nucleation points, around which non-thermoresponsive bilayers (containing the azide-functionalized lipid) were encouraged to assemble, simultaneously encapsulating the enzyme in the intermembrane space. A control was included, in which no second bilayer was generated. Each sample (700 μl) was immediately treated with a Trypsin solution (0.25 wt%) to degrade unencapsulated β-Gal. Enzyme activity at the same concentration was assessed with and without trypsin addition (see Supplementary Fig. 10 for bulk assay). Samples were analysed using a CARY ultraviolet–visible/fluorescence spectrometer at both room temperature and 42 °C, tracking the emergence of a fluorescent signal at λ_em_ = 517 nm over time, signifying hydrolysis of the substrate to fluorescein.

## Online content

Any methods, additional references, Nature Portfolio reporting summaries, source data, extended data, supplementary information, acknowledgements, peer review information; details of author contributions and competing interests; and statements of data and code availability are available at 10.1038/s41557-024-01584-z.

## Supplementary information

Supplementary Information Supplementary Figs. 1–13.

Supplementary Data 1 Source data for Supplementary Figs. 4, 6, 8 and 9–12.

## Source data

Source Data Fig. 2 Source data for DLS Fig. 2a.

Source Data Fig. 3 Source data for histogram Fig. 3b.

Source Data Fig. 4 Source data for percent calcein release Fig. 4b.

Source Data Fig. 5 Source data for multi-stage release Fig. 5b,c.

Source Data Fig. 6 Source data for β-galactosidase assay Fig. 6b,c.

## Data Availability

All data pertaining to the main article and Supplementary Information are available in the Source Data. Source Data are provided with this paper.
