# Supplementary material for: Engineering a nanoscale liposome-in-liposome for in situ biochemical synthesis and multi-stage release
Source: Nat Chem. 2024 Jul 15;16(10):1612–20. doi: 10.1038/s41557-024-01584-z (PMC11446840; doi:10.1038/s41557-024-01584-z)
Supplement: Supplementary file 1 — Supplementary Figs. 1–13. [file 41557_2024_1584_MOESM1_ESM.pdf]

nature chemistry

Supplementary information https://doi.org/10.1038/s41557-024-01584-z

Engineering a nanoscale liposome-in-liposome for in situ biochemical synthesis and multi-stage release

In the format provided by the authors and unedited

Engineering a nanoscale liposome-in-liposome for in situ biochemical synthesis and multi-stage release

Colin P. Pilkington,[1,2] Ignacio Gispert,[2] Suet Y. Chui,[2] John. M. Seddon [1] and Yuval Elani [2*]

1. Department of Chemistry, Molecular Science Research Hub, Imperial College London, 82 Wood Lane, London, W12 0BZ, UK

2. Department of Chemical Engineering, Exhibition Road, Imperial College London, London, SW7 2AZ, UK

*To whom correspondence may be addressed. E-mail: y.elani@imperial.ac.uk

Supplementary information

Source data files are provided where appropriate

SI Figure 1: Additional micrographs of unilamellar liposomes and concentrisomes

SI Figure 2: Representative micrographs of bicelles

SI Figure 3: Representative micrographs of system without click chemistry

SI Figure 4: Azidocoumarin assays

SI Figure 5: Micrographs of concentrisomes with different linker lengths

SI Figure 6: Calcein release assay controls

SI Figure 7: Composition table

SI Figure 8: Multi-stage release controls

SI Figure 9: Encapsulation efficiencies

SI Figure 10: Trypsin control

SI Figure 11: Collection of fluorescein product

SI Figure 12: Two separate liposome populations (Beta-Gal. control)

SI Figure 13: CAD drawing of chip

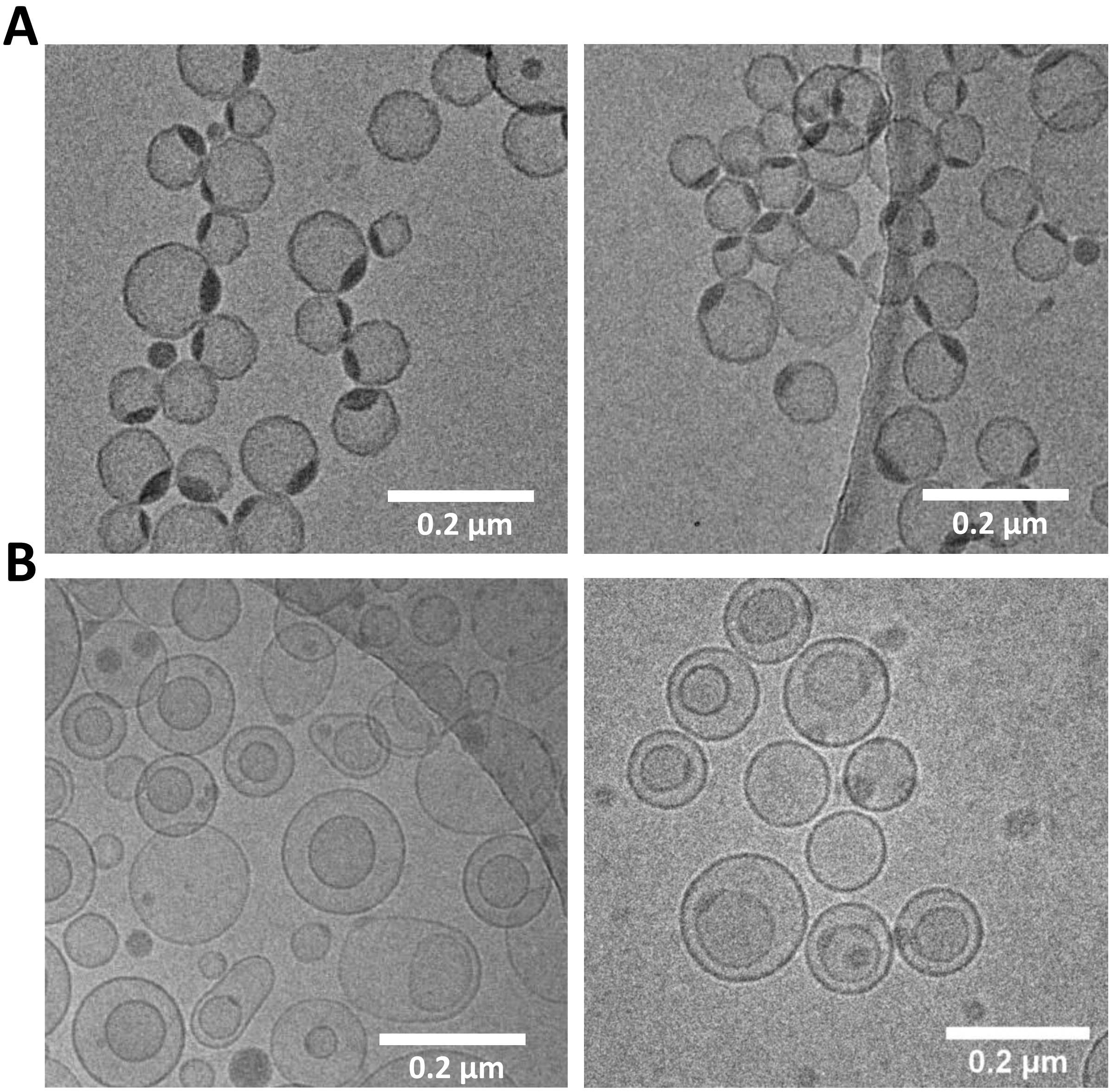

SI Figure 1 (A) *Additional cryo-TEM images of unilamellar liposomes (DBCO functionalised*) (**B**) Additional *cryo-TEM images of concentrisomes (**B**). The darker, electron dense domain-like portions are hypothesised to be cholesterol-rich lipid droplets.*

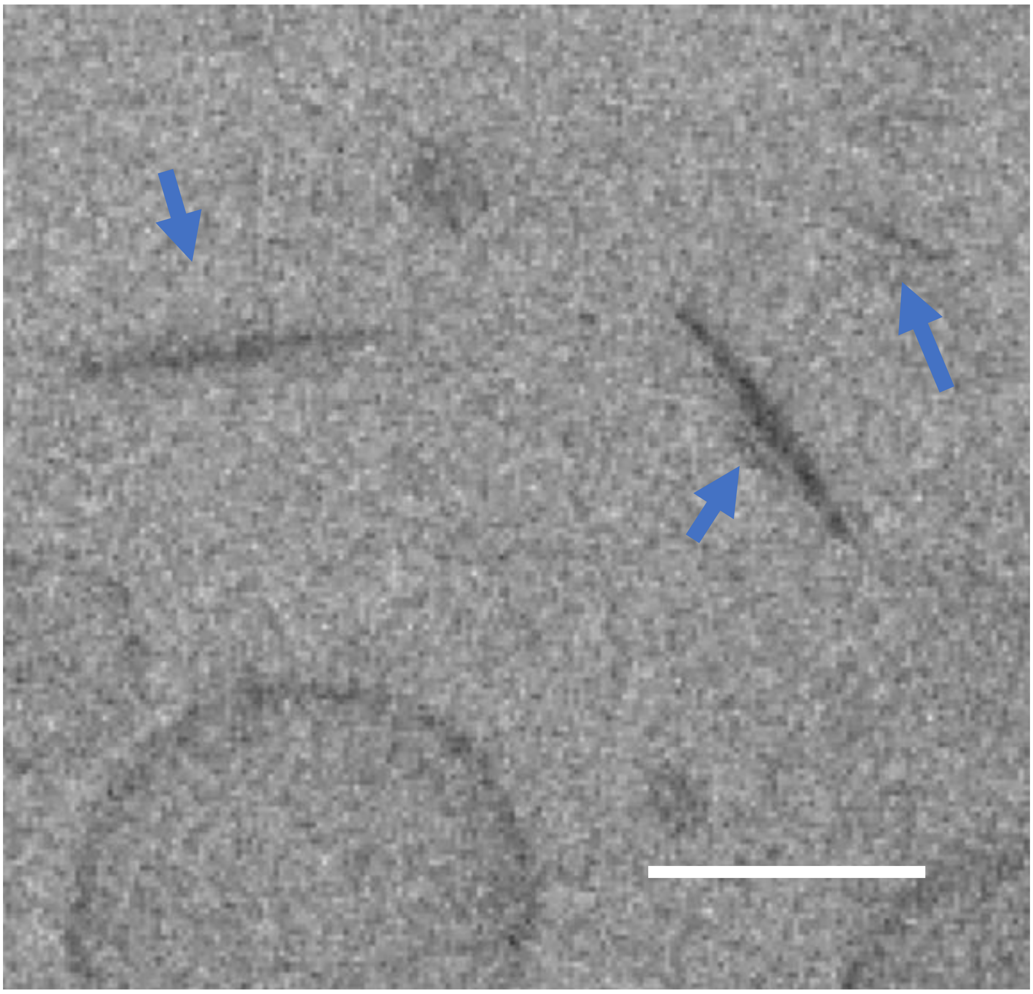

SI Figure 2: *Representative cryo-TEM image of nanodisks/bicelles (marked with blue arrows. These stabilised bilayer disks (here viewed side-on) known to be a common side-product in DPPC/DSPE-PEG binary mixtures. Scale bar indicates 75 nm.*

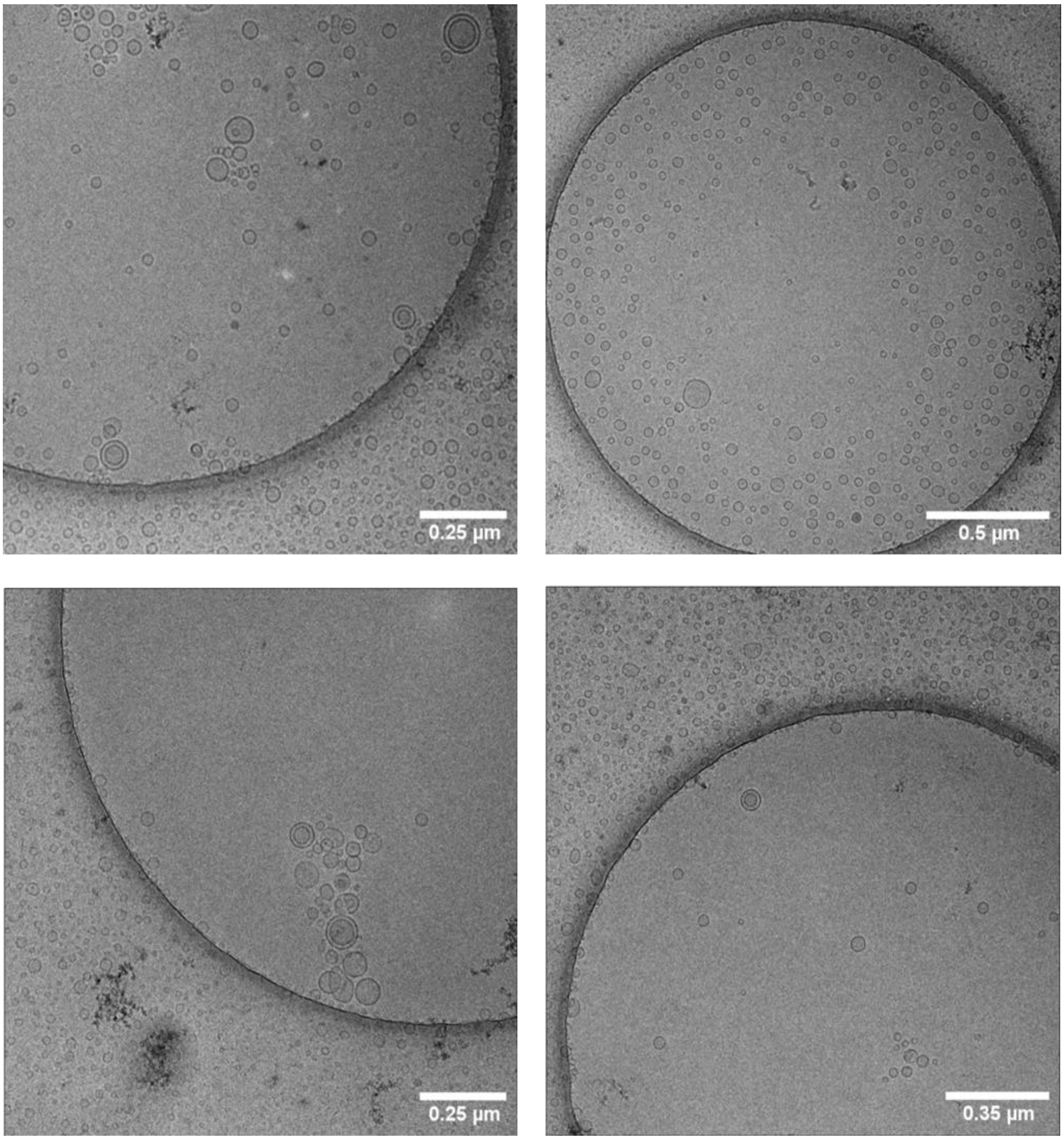

SI Figure 3: *Representative cryoTEM images of double bilayer vesicles and SUVs generated where no terminal azides or DBCO groups are present. Composition of inner and outer bilayers are DPPC:Cholesterol:DSPE-PEG$_{2k}$ (53:42:5 mol%; 6.8 mM). <10 % of total particles counted had more than one bilayer. Crucially however, MLVs never exceeded bilamellarity (ie. only double bilayer vesicles were observed).*

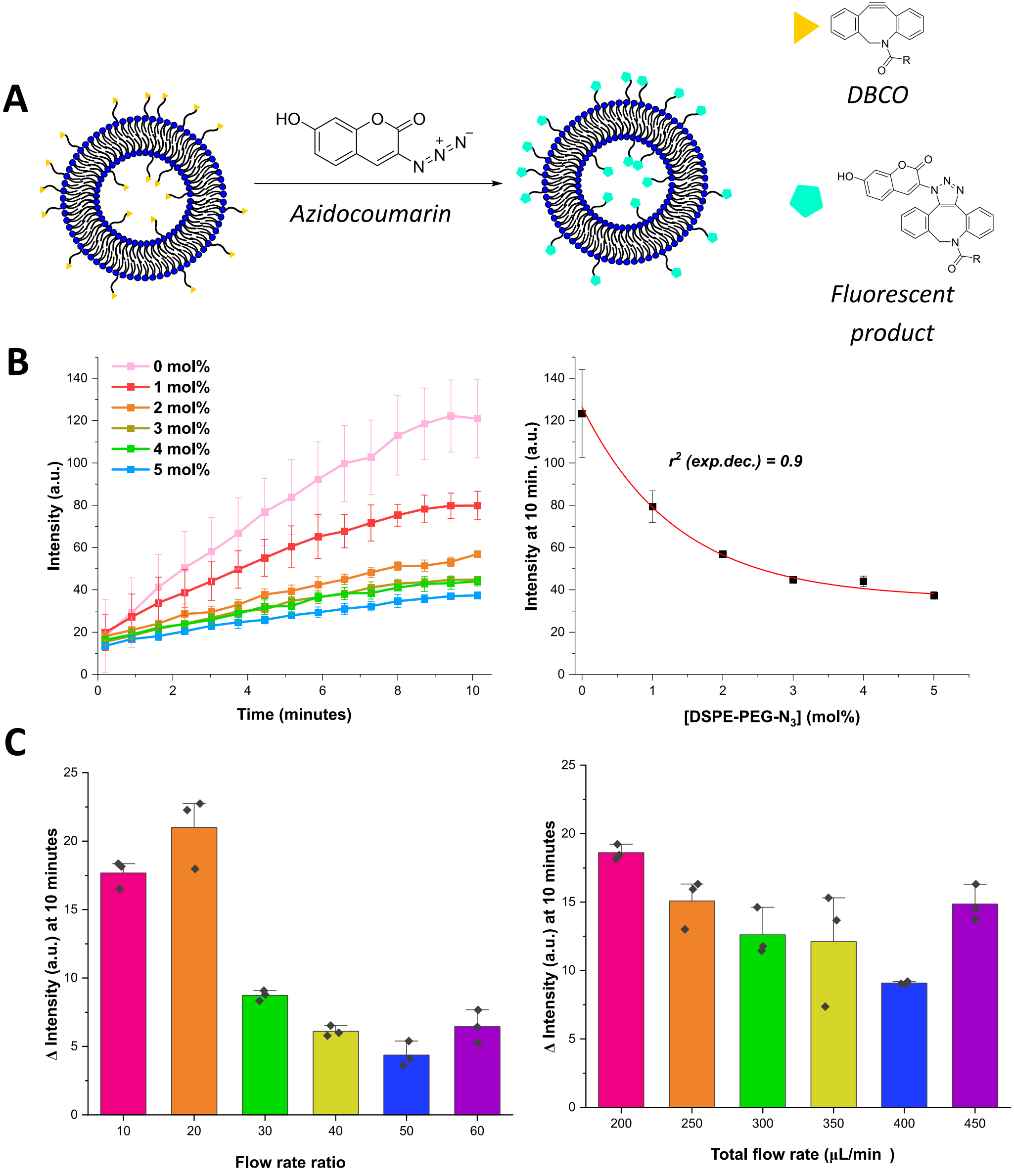

SI Figure 4. ***A.*** *A graphic illustrating the reaction between DBCO-functionalised vesicles and the fluorogenic dye azidocoumarin. An otherwise non-fluorescent molecule, the triazole adduct produces a fluorescent signal, here detected with λ_{abs} = 404 nm and λ_{em} = 477 nm.* ***B.*** *Fluorescence intensity traces for DBCO vesicle populations exposed to a secondary lipid composition with varying mol% of DSPE-PEG$_{2K}$-N$_3$. Each was treated with the same amount of azidocoumarin (6.15 µM). Error bars indicate the standard deviation about the mean for three separate experiments. Results indicated that DBCO moieties present on the surface of pre-formed vesicles took part in SPAAC with opposing bilayers during concentrisome formation. An exponential decay fit for fluorescence intensity values at 10 minutes.* ***C.*** *Optimising microfluidic flow conditions using the azidocoumarin assay. Flow rate ratio (FRR) is shown on the left, and total flow rate (TFR) is shown on the right. Error bars indicate the standard deviation about a mean for ΔI in three separate experiments (see main text for definitions).*

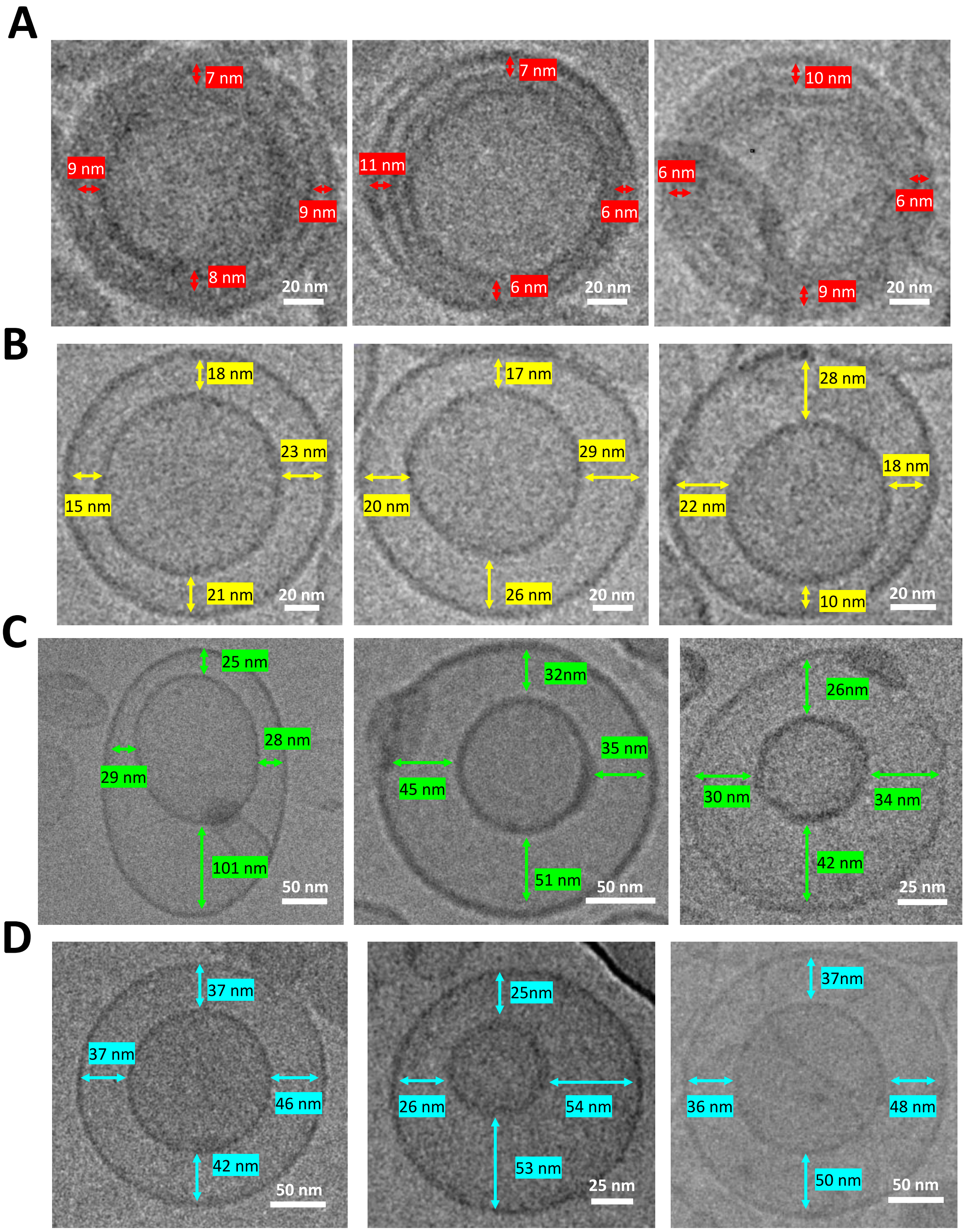

SI Figure 5: *Additional micrographs of double-bilayer vesicles made using click chemistry. Note that values can vary depending on the position of measurement. As such, an average value for* d_{inter} *was taken, equal to the average of four values per-particle, each at 90 ° with respect to one another.* ***A*** *= No linker,* ***B*** *= PEG (2K) & PEG (2K)* ***C*** *= PEG (2K) & PEG (5K),* ***D*** *= PEG (5K) & PEG (5K).*

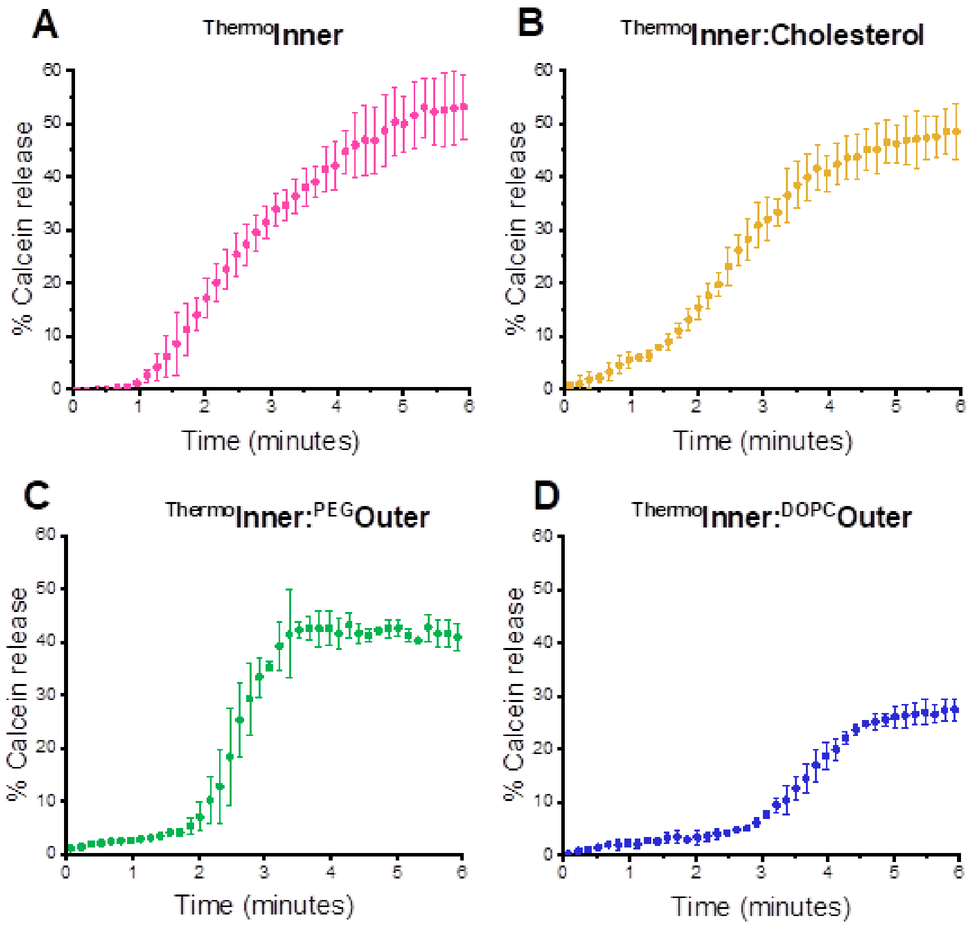

SI Figure 6. *Percent calcein release profiles at 42 °C for each composition tested, the details of which can be found in the main text and SI Figure 7. Values were calculated from fluorescence intensities after addition of surfactant (Triton X-100 5 wt% 2.5 µL/min). Error bars indicate ± the standard deviation about the mean percent release for three seperate experiments.* ***A****. Release profile for liposomes containing calcein, co-flowed in an MHF chip with ethanol only in the central stream. This profile served as a benchmark for all other release profiles.* ***B****. Release profile for liposomes co-flowed alongside a solution of cholesterol (2.86 mM in ethanol). The release profile resembled that of free liposomes, suggesting that cholesterol did not insert into the liposome bilayer (enough to alter the transition temperature).* ***C****. The release profile for liposome co-flowed alongside a solution of DPPC:Cholesterol:DSPE-PEG$_{2K}$ (53:42:5 mol% in ethanol). This control was used to investigate the necessity of click chemistry in the release of calcein, and by extension, the formation of concentrisomes.* ***D****. Liposomes were co-flowed alongside a solution of DOPC:DSPE-PEG$_{2K}$-N$_3$ (95:5 mol%). A reduction in percent calcein release was observed, suggesting that a second non-thermo-responsive bilayer had successfully assembled around thermo-responsive liposomes via click chemistry.*

Sample	Inner composition	Outer composition
^{Thermo}*Inner*	DPPC:Cholesterol:DSPE-PEG$_{2K}$-DBCO (90:5:5 mol%)	Ethanol only
^{Thermo}*Inner:*$^{Non\text{-}thermo}$*Outer*	DPPC:Cholesterol:DSPE-PEG$_{2K}$-DBCO (90:5:5 mol%)	DPPC:Cholesterol:DSPE-PEG$_{2K}$-N_3 (53:42:5 mol%)
^{Thermo}*Inner:*^{Thermo}*Outer*	DPPC:Cholesterol:DSPE-PEG$_{2K}$-DBCO (90:5:5 mol%)	DPPC:Cholesterol:DSPE-PEG$_{2K}$-N_3 (90:5:5 mol%)
^{Thermo}*Inner:Cholesterol*	DPPC:Cholesterol:DSPE-PEG$_{2K}$-DBCO (90:5:5 mol%)	Cholesterol (2.86 mM)
^{Thermo}*Inner:*^{PEG}*Outer*	DPPC:Cholesterol:DSPE-PEG$_{2K}$-DBCO (90:5:5 mol%)	DPPC:Cholesterol:DSPE-PEG$_{2K}$ (53:42:5 mol%)
^{Thermo}*Inner:*^{DOPC}*Outer*	DPPC:Cholesterol:DSPE-PEG$_{2K}$-DBCO (90:5:5 mol%)	DOPC:DSPE-PEG$_{2K}$-N_3 (95:5 mol%)

SI Figure 7: *Table indicating the precise composition of each successive bilayer in concentrisomes. The table is most applicable to the assay described in SI Figure 6 and Figure 4 in the main text.*

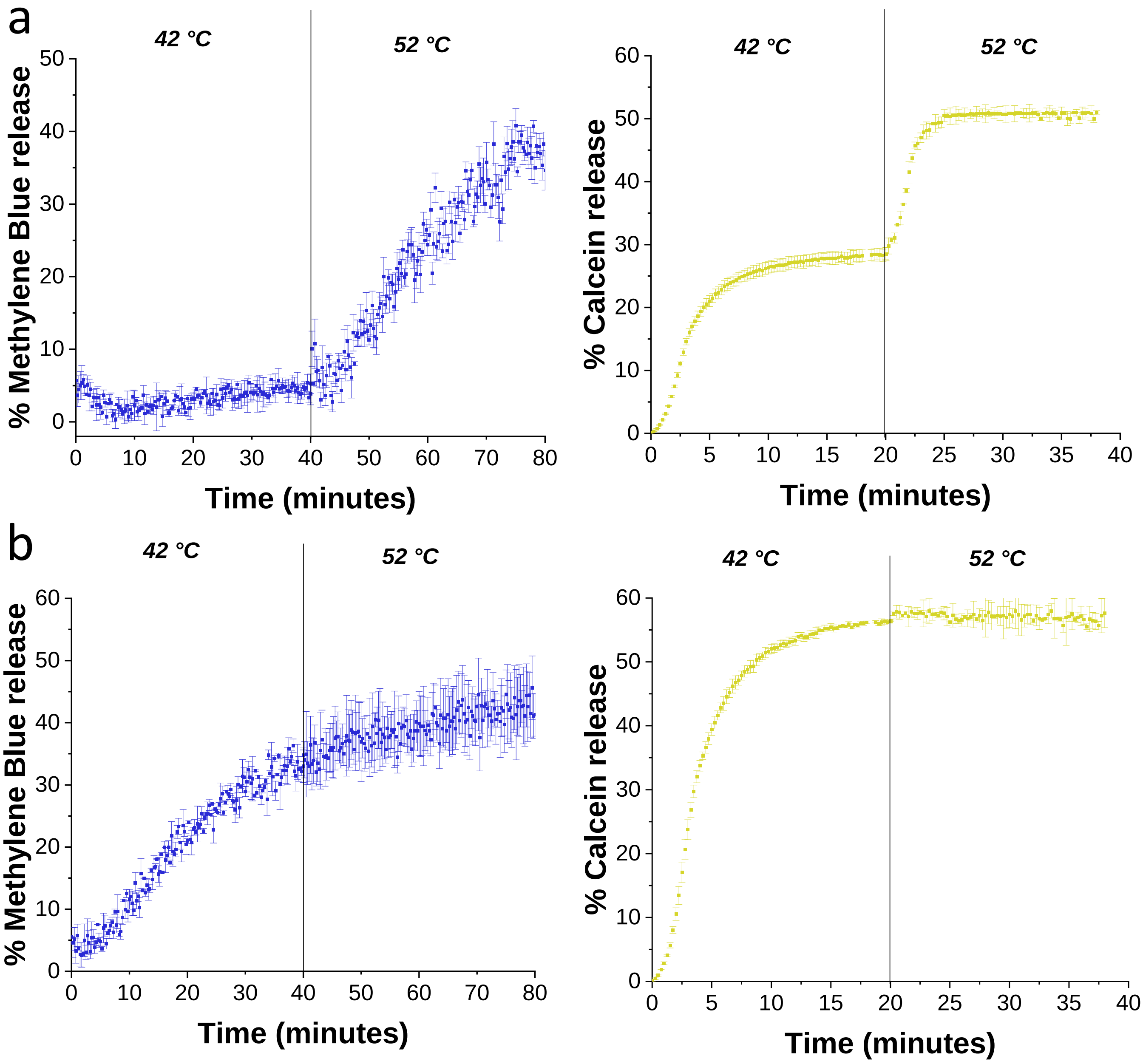

SI Figure 8: *Additional controls to support results discussed in Figure 5 of the manuscript, where calcein and methylene blue are released sequentially.* ***A****. Calcein was encapsulated in the inner compartment of a concentrisome system, and methylene blue in the intermembrane space. The inner bilayer was composed of a DPPC composition (T_m = 42 °C) and the outer bilayer composed of a DSPC composition (T_m = 52 °C). Both dyes were released at 52 °C. Some calcein release at the lower transition temperature was thought to arise from unilamellar liposomes that had not undergone click chemistry. Data are represented as mean values ± 1 standard deviation for n = 3.* ***B****. Calcein and methylene blue were encapsulated as in A, this time with both bilayers composed of a DPPC composition (T_m = 42 °C). Both dyes were observed to release at 42 °C. Data are represented as mean values ± 1 standard deviation for n = 3.*

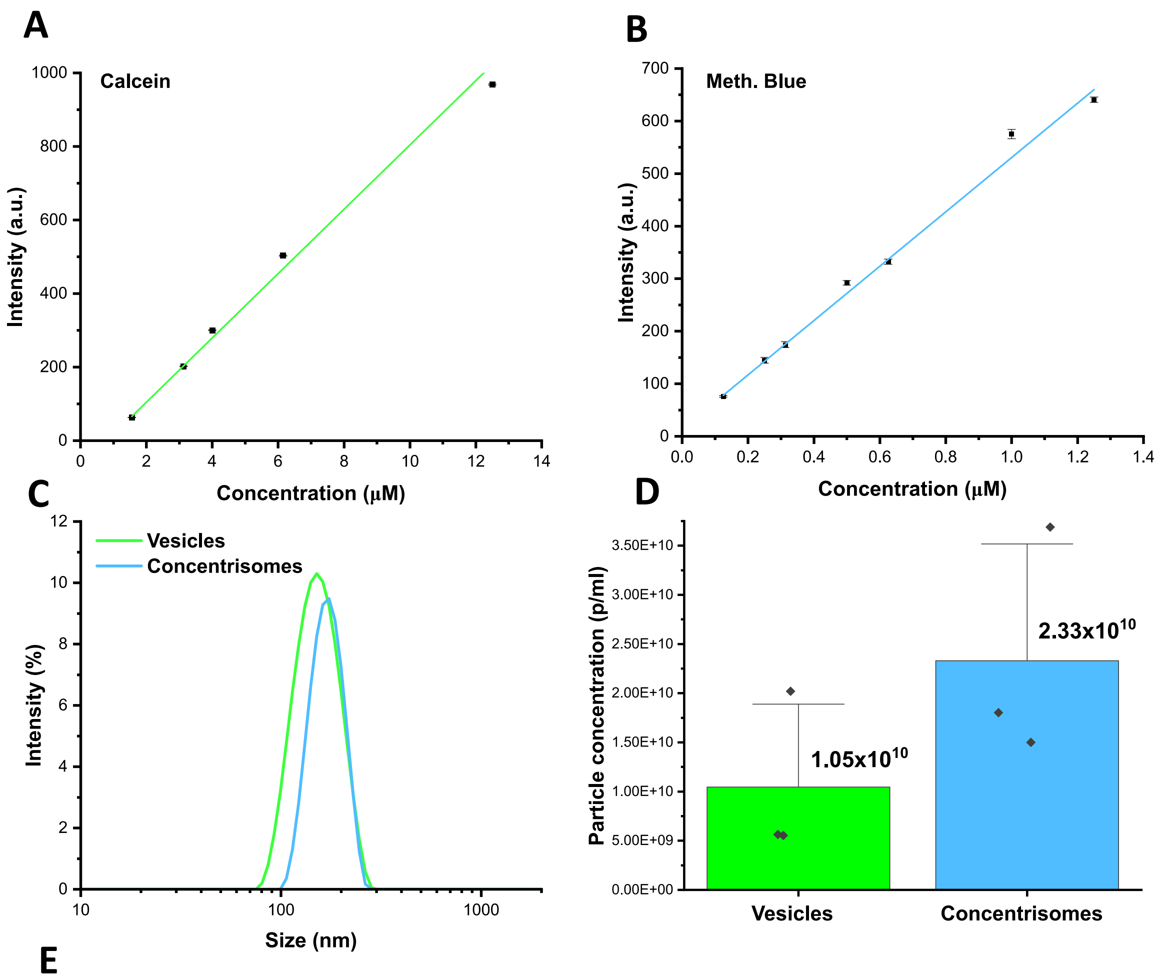

E

Sample	Size	PDI	Particles/ml	[Lipid]*	[Input dye]	[Dye after lysis]	% Encapsulation
Vesicles	150 nm	0.16	1.046×10^{10}	0.06 mM	40 mM	5.040 ± 0.080 μM	1.250×10^{-2}
Concentrisomes	180 nm	0.26	2.030×10^{10}	0.06 mM	40 mM	0.580 ± 0.005 μM	1.260×10^{-3}

SI Figure 9: *Determination of the approximate encapsulation efficiency for liposomes (composed of $^{Non\text{-}Thermo}$Inner) with calcein, and concentrisomes (composed of $^{Non\text{-}Thermo}$Inner:^{Thermo}Outer) with methylene blue encapsulated in the inter-membrane space. The same system was used for the multi-stage release assay.* ***A.*** *Calibration curve for calcein. The intensity value of calcein-loaded vesicles AFTER lysis was compared to the linear fit shown here, and used to calculate the approximate concentration of calcein released.* ***B.*** *Calibration curve for methylene blue. The same method was used to calculate released dye concentration as in A. For both A and B data are represented as mean values ± 1 standard deviation for n = 3.* ***C.*** *MADLS derived distributions for $^{Non\text{-}Thermo}$Inner liposomes (green) and $^{Non\text{-}Thermo}$Inner:^{Thermo}Outer concentrisomes (blue).* ***D.*** *MADLS derived particle concentration estimates (in particles/ml) for both populations. Error bars indicate the deviation about an average concentration for n=3.* ***E.*** *A summary of all data pertaining to the encapsulation efficiency for vesicles and the inter-membrane space of concentrisomes. Note: lipid concentrations are a rough estimate as nanoparticle populations were subject to size exclusion chromatography.*

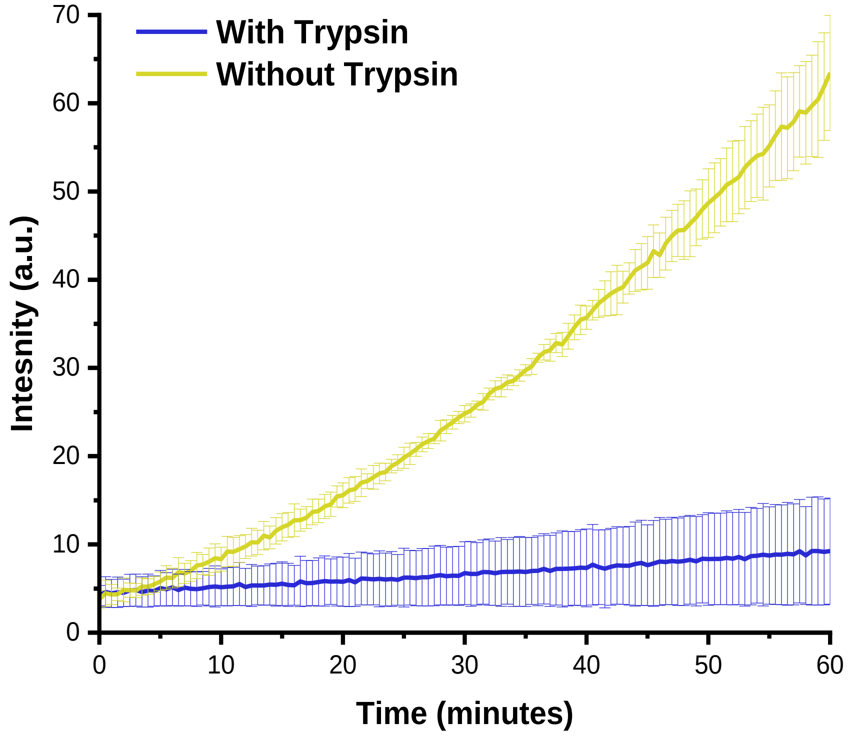

SI Figure 10. *A control experiment studying the effects of trypsin (0.025 wt%) on β-galactosidase (0.5 Uml*$^{-1}$*) hydrolase activity with fluorescein Di-β-D-galactopyranoside (0.15 mM) at room temperature. Trypsin protease (0.25 wt%) was added directly to a cuvette containing 450 µL of a mixture of β-galactosidase and fluorescein Di-β-D-galactopyranoside, then immediately measured for fluorescence (*λ_{ex} *= 498 nm;* λ_{em} *= 517 nm). Alongside this was measured a cuvette of the same composition excluding trypsin (buffer (50 µL) added in its place (yellow trace). Data are represented as mean values ± 1 standard deviation for n = 3.*

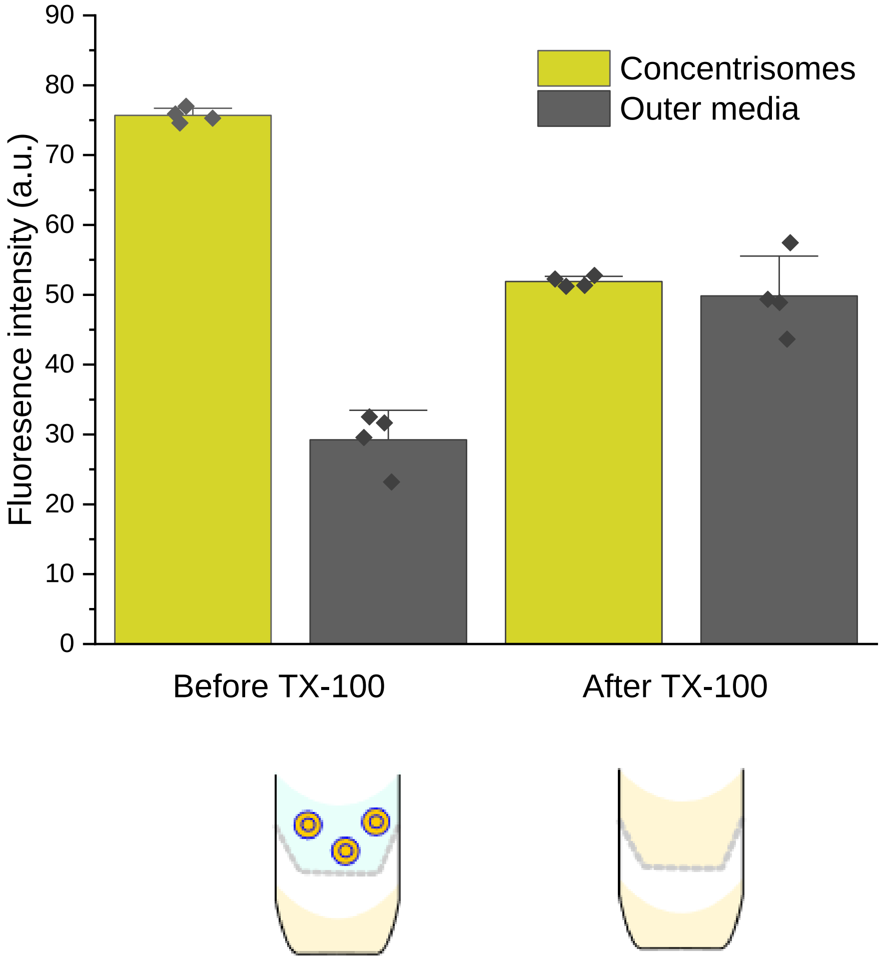

SI Figure 11. *Fluorescence intensities of concentrisome after beta-galactosidase mediated hydrolysis of FDG (λex = 498 nm; λem = 517 nm) and concentration using Amicon centrifugal filter units (mW cut off 10 kDa). The results indicated the majority of produced fluorescein is retained in the concentrisome system. Upon treatment with surfactant, fluorescein is released into the bulk and passes freely through the filter. Data are represented as mean values ± 1 standard deviation for n = 4.*

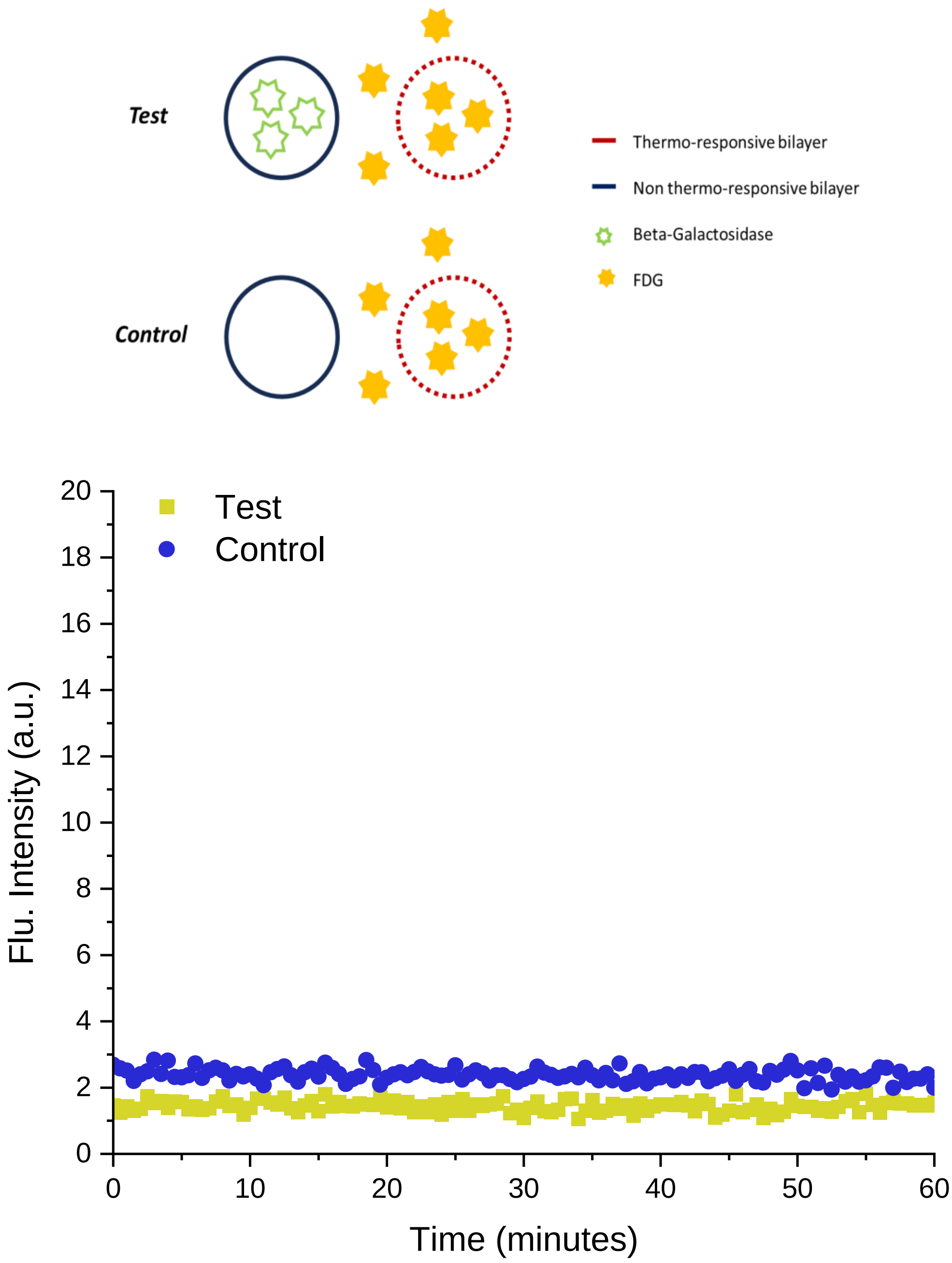

SI Figure 12. *Fluorescence intensities of concentrisome after beta-galactosidase mediated hydrolysis of FDG (λ_{ex} = 498 nm; λ_{em} = 517 nm) and concentration using Amicon centrifugal filter units (mW cut off 10 kDa). The results would indicate the majority of produced fluorescein is retained in the concentrisome system. Upon treatment with surfactant, fluorescein is released into the bulk and passes freely through the filter.*

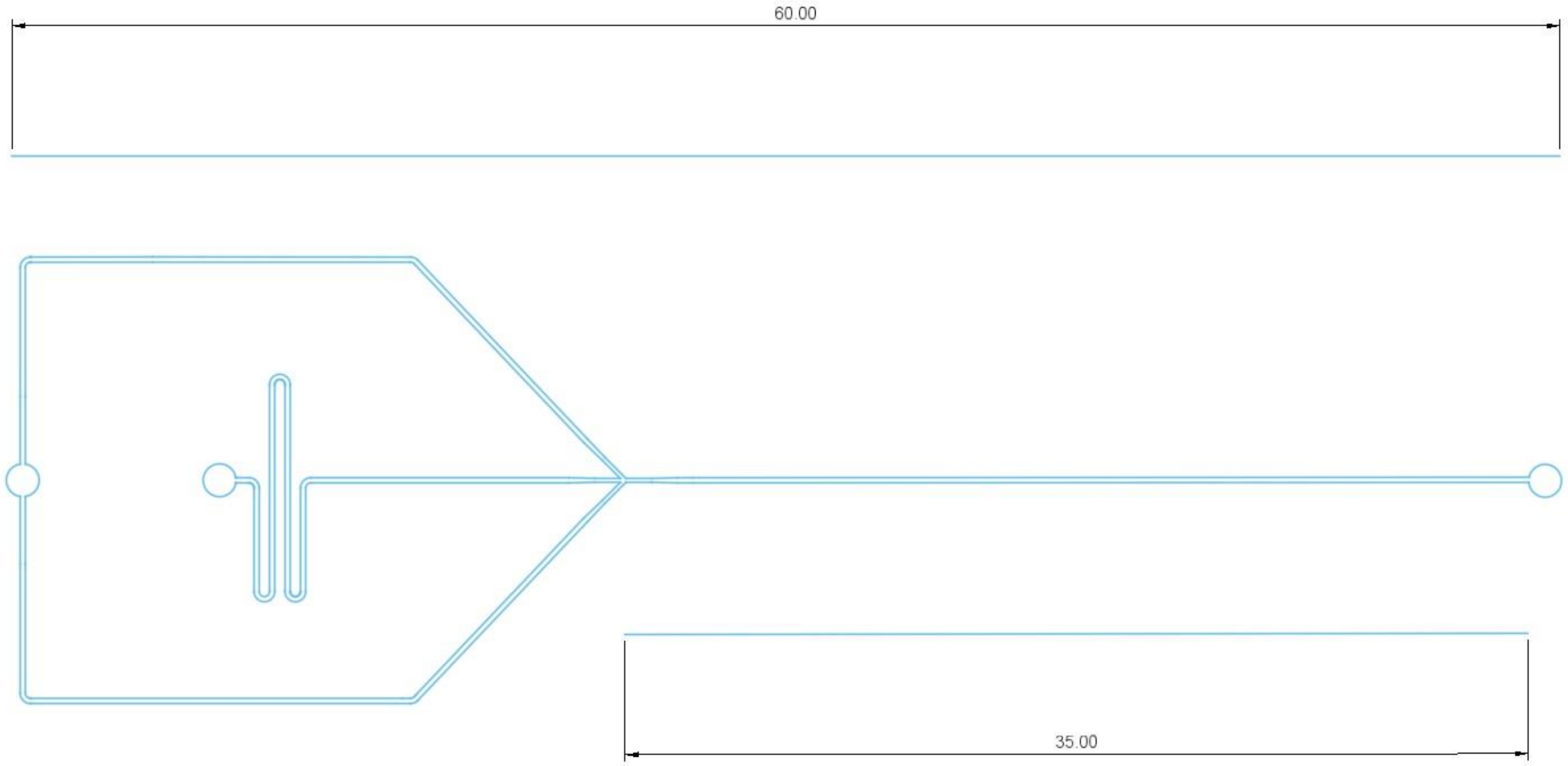

SI Figure 13: *Snapshot of CAD filed used to generate the microfluidic chip capable of MHF. The CAD file is available by request from the corresponding author. Values are in millimetres.*
